# SIRT1 Activation Suppresses Corneal Endothelial–Mesenchymal Transition via the TGF-β/Smad2/3 Pathway

**DOI:** 10.3390/cimb46120827

**Published:** 2024-12-06

**Authors:** Yi Yu, Ruilin Guo, Jie Ling, Chenjia Xu, Minglu Ma, Xiaojuan Dong, Jing Wu, Ting Huang

**Affiliations:** State Key Laboratory of Ophthalmology, Zhongshan Ophthalmic Center, Sun Yat-sen University, Guangdong Provincial Key Laboratory of Ophthalmology and Visual Science, Guangzhou 510060, China; yuyi57@mail2.sysu.edu.cn (Y.Y.); linlin128@126.com (R.G.); lingjie2018@163.com (J.L.); xuchj1189@163.com (C.X.); mingluma@126.com (M.M.); qingerdong@126.com (X.D.); wujing09@hotmail.com (J.W.)

**Keywords:** SIRT1, endothelial–mesenchymal transition, corneal endothelial cells, TGF-β/Smad2/3

## Abstract

Endothelial–mesenchymal transition (EnMT) is the transversion of endothelial cells to mesenchymal cells under certain physiological or pathological conditions. When EnMT occurs in the corneal endothelium, corneal endothelial cells (CECs) lose their normal function and thus cannot maintain corneal clarity. Studies have shown that the mechanism of EnMT in CECs involves the transforming growth factor-β (TGF-β) signaling pathway, and one of the important inhibitors of the TGF-β/Smad2/3 pathway is sirtuin-1 (SIRT1). In this study, we used a rat model of corneal endothelium injury and TGF-β1-treated human CECs to induce EnMT, aiming to explore whether SIRT1 activation inhibits corneal EnMT in vivo and in vitro. SIRT1 was activated and suppressed using resveratrol (RSV) and EX527, respectively. The endothelial markers and mesenchymal markers were measured by immunofluorescence and Western blot assays. Co-immunoprecipitation was used to detect the interaction between SIRT1 and Smad2/3. The results showed that after mechanical injury, the group treated with RSV-activated SIRT1 regained corneal transparency and recovered from edema faster than the control group. Moreover, RSV-activated SIRT1 downregulated the expression levels of alpha smooth muscle actin (α-SMA), vimentin, and Snail and upregulated the expression levels of E-cadherin and Na^+^/K^+^-ATPase both in vivo and in vitro, but these effects were reversed when SIRT1 was inhibited by EX527. SIRT1 also upregulated the expression levels of TGF-β receptor 1 and phosphorylated Smad2/3. The interaction between SIRT1 and Smad2/3 in vitro was confirmed by co-immunoprecipitation. Overall, our results indicate that SIRT1 activation inhibits corneal EnMT via the TGF-β/Smad2/3 pathway, which may be a potential therapeutic target for corneal endothelium dysfunction.

## 1. Introduction

Endothelial–mesenchymal transition (EnMT) refers to the transversion or differentiation of endothelial cells into mesenchymal cells under certain pathological or physiological conditions, and this phenomenon is accompanied by phenotypic and functional changes in endothelial cells [1]. Endothelial cells become spindle-shaped [2], and they lose their endothelial function markers (e.g., Na^+^/K^+^-ATPase) and cell adhesion molecules (e.g., E-cadherin), obtaining mesenchymal-specific markers (e.g., alpha smooth muscle actin (α-SMA), vimentin, and Snail) instead [3,4]. EnMT plays a pivotal role in injury repair and is widely involved in various pathological processes, such as tumor invasion and fibrosis of the heart, lung, kidney, eye, and other organs [5,6].

During corneal injury, EnMT usually occurs in the part of the corneal endothelium that remains intact [7]. In this process, corneal endothelial cells (CECs) undergo morphological changes and fibrotic transformation [8]. Moreover, CECs lose their normal pump function, leading to edema and corneal opacity [9,10]. The occurrence of EnMT is induced by various extracellular signals or factors, of which TGF-β signaling is a major EnMT inducer [11] wherein TGF-β transduces signals from receptors to the nucleus via the activation of Smad molecules [12]. TGF-β is an important cytokine that regulates tissue inflammation as well as repairs damages [13]. Due to the chemotactic effects of TGF-β, fibroblasts migrate to the site of injury and become activated, resulting in the increased synthesis and secretion of the extracellular matrix (ECM) [14].

Sirtuin1 (SIRT1) is a cell-metabolic coenzyme NAD+-dependent histone deacetylase (HDAC) [15,16]. It has been proved in many studies that SIRT1 has the effect of prolonging the lifespan of lower organisms and delaying the development of various age-related diseases [17,18,19]. SIRT1 is expressed in the nuclei and cytoplasm of cells in most ocular tissues, including the cornea, iris, lens, and retina [20]. Moreover, SIRT1 expression plays an essential role in the development and prognosis of multiple ophthalmic diseases [21], such as cataract [22] and diabetic retinopathy [23]. However, the role of SIRT1 in corneal EnMT remains poorly studied. Therefore, we investigated the role of SIRT1 in corneal EnMT and its probable mechanism, hoping to find new potential therapeutic targets for corneal diseases.

## 2. Materials and Methods

### 2.1. Animals

Adult male Sprague Dawley (SD) rats weighing 200–250 g and 6–8 weeks of age were purchased from Guangzhou Southern Medical University Experimental Animal Technology Co., Ltd. (Guangzhou, China). All animal procedures were performed in accordance with the Guide for the Care and Use of Laboratory Animals published by the US National Institutes of Health. The experimental procedures were approved by the Institutional Animal Care and Use Committee of the Zhongshan Ophthalmic Center (Approval No. O2023016).

### 2.2. Cell Culture and Treatment

The human CEC (HCEC) line B4G12 was cultured in a human endothelial medium (Invitrogen, Waltham, MA, USA) containing 2.5% fetal bovine serum (FBS; Gibco, Waltham, MA, USA) and 10 ng/mL human basic fibroblast growth factor (FGF; Beyotime, Shanghai, China). To induce EnMT, we cultivated some cells in a medium with 10 ng/mL of recombinant TGF-β1 (MedChemExpress, Monmouth Junction, NJ, USA) [4]. In the treatment groups, the HCECs were randomly assigned for treatment with resveratrol (RSV) (20 μM) or with RSV (20 μM) + EX527 (10 μM) [16]. The medium was changed every two days, and the cultures were incubated at 37 °C in 5% carbon dioxide.

### 2.3. Animal Model and Treatment

**Figure 1:**
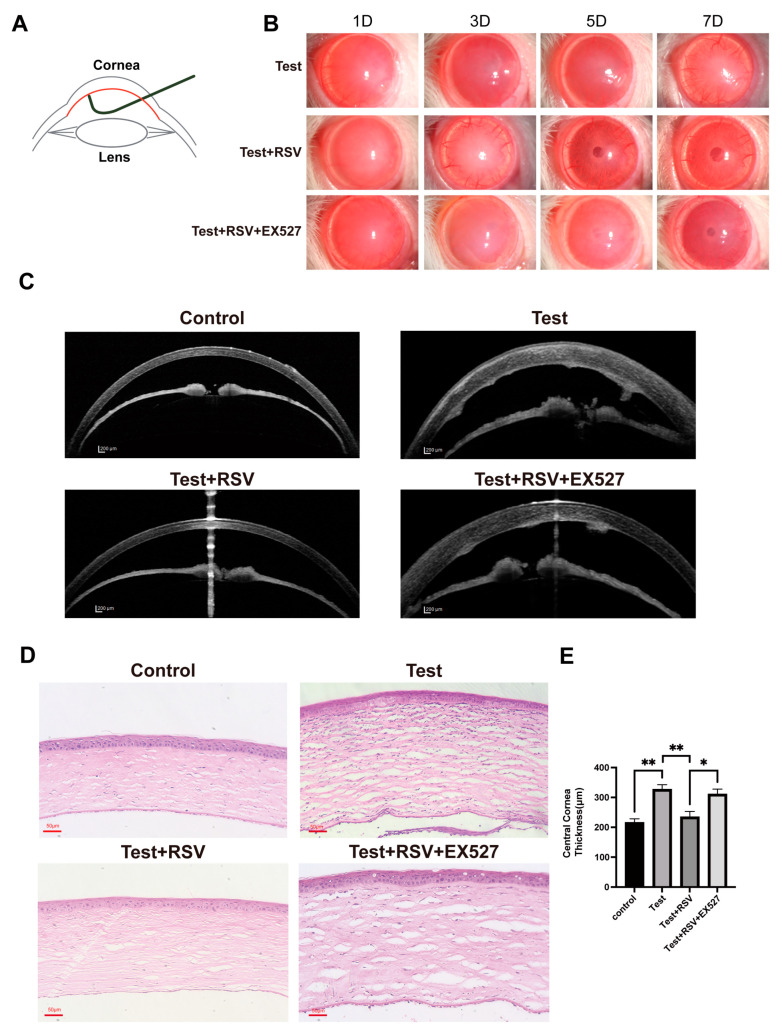
Effects of SIRT1 activation on corneal clarity and central corneal thickness in injury-induced EnMT rat model. Endothelial layer is indicated in red. (**A**) Diagram of the establishment of the corneal endothelial injury model. (**B**) Changes in corneal clarity in rats as observed under a slit lamp microscope on days 1, 3, 5, and 7 after injection with RSV or RSV + EX527. (**C**) AS-OCT images of central cornea. Scale bar = 200 μm. AS-OCT: anterior segment optical coherence tomography. (**D**) H&E staining of the cornea. Scale bar = 100 μm. H&E: hematoxylin and eosin. (**E**) Quantification of the central cornea thickness. * *p* < 0.05, ** *p* < 0.01, *n* = 3.

The rats were anesthetized with 1% pentobarbital sodium (40 mg/kg via intramuscular injection) and then divided into four groups (18 per group): control, Test, Test + RSV, and Test + RSV + EX527. In the Test group, the corneal endothelium of the right eye was mechanically scraped using a bent 32-gauge needle (Inami, Tokyo, Japan) from the anterior chamber to mimic corneal endothelial damage (Figure 1A). The procedures were carefully performed under an operating microscope to avoid causing any damage to the lens and iris. RSV, a potent SIRT1 agonist, was first dissolved with DMSO (15 mg/mL) as a mother liquid and then diluted with normal saline to 5 mg/mL. In the Test + RSV group, 20 mg/kg/day RSV was injected intraperitoneally following mechanical scraping of the corneal endothelium. The Test + RSV + EX527 group received intraperitoneal injections of RSV (20 mg/kg/day) and EX527 (5 mg/kg/day) [16]. In the control and Test groups, an equivalent solvent was injected as contrast.

All treatments were performed daily for 7 consecutive days after modeling. A slit lamp microscope (Topcon, Tokyo, Japan) was used to observe corneal clarity on days 1, 3, 5, and 7. An AS-OCT (anterior segment optical coherence tomography) was used to detect the thickness of the central cornea in vivo on day 7.

### 2.4. Cell Viability and Migration Assays

Cell viability was evaluated using a cell counting kit-8 (CCK-8; Dojindo, Kumamoto, Japan). Cells were seeded in a 96-well plate at 1 × 10^3^ cells/well and allowed to grow for 24 h with different treatments (control, TGF-β1, RSV, TGF-β1 + RSV, EX527 and TGF-β1 + RSV + EX527). Then, the CCK-8 solution was added to the plates. After 2–3 h incubation, optical density was quantified at 450 nm using a microplate reader (BioTek, Winooski, VT, USA).

The effect of SIRT1 on the migration of TGF-β1-induced HCECs was evaluated by a scratch wound assay. In brief, cells were seeded on a 24-well plate at 5 × 10^4^ cells/well and allowed to grow for 48 h to reach 90% confluence. The cell monolayer was scratched using a scratcher with a 24-well lid (SPLScar Scratcher, SPL Life Sciences, Pocheon-si, Republic of Korea) and then washed with a phosphate buffer solution (PBS) three times. The cells were subsequently incubated with different treatments (control, TGF-β1, TGF-β1 + RSV, and TGF-β1 + RSV + EX527) for 12 h. The migration of HCECs into the wound area was observed using an inverted microscope.

### 2.5. Histological Quantification of Corneal Endothelial Dysfunction

Corneal clarity was evaluated using a camera-equipped slit lamp microscope. After 7 days of treatment, the rats were sacrificed; their whole eyeballs were immediately fixed with 4% paraformaldehyde (PFA; Bioss, Beijing, China) and then dehydrated and embedded in paraffin. Five micrometer-thick transverse sections of the eyeballs revealing a transverse view of the cornea were prepared and subsequently deparaffinized. Finally, the slices were stained with hematoxylin and eosin (H&E) for histological observation.

### 2.6. Immunofluorescence Staining

The rats were sacrificed by overdose of anesthesia after 7 days of treatment. Full-thickness rat corneas were quickly excised and fixed with 4% PFA for 30 min at room temperature and then penetrated with 0.5% Triton X-100 for 15 min. After being washed with PBS three times, the samples were blocked with 5% bovine serum albumin (BSA, Bioss, China) for 60 min at room temperature. Then, the samples were co-incubated with primary antibodies against SIRT1 (1:200, Proteintech, San Diego, CA, USA) and with α-SMA (1:200, Cell Signaling Technology, Danvers, MA, USA) at 4 °C overnight. The corneal samples were subsequently stained with a Goat anti-Mouse IgG (H+L) Highly Cross-Adsorbed Secondary Antibody (Alexa Fluor Plus 488; Invitrogen, Carlsbad, CA, USA) and a Goat anti-Rabbit IgG (H+L) Highly Cross-Adsorbed Secondary Antibody (Alexa Fluor Plus 594, 1:200; Invitrogen) for 2 h at room temperature, and the cell nuclei were counterstained with 4,6-diamidino-2-phenylindole (DAPI; Elabscience, Wuhan, China). The corneal endothelial layer was carefully torn off, and then a fluorescence microscope (Carl Zeiss Meditec, Oberkochen, Germany) was used for observation and for capturing images.

For the in vitro experiment, the HCECs were first washed with PBS three times to clean the medium and then fixed with 4% PFA for 20 min at room temperature. After being washed again with PBS three times, the cells were treated with 0.5% Triton X-100 for 15 min at room temperature. BSA (5%) was used for permeabilization for 30 min, and then the cells were co-incubated with antibodies against SIRT1 (1:200, Proteintech, San Diego, CA, USA) and α-SMA (1:200, Cell Signaling Technology, USA) at 4 °C overnight. The cells were subsequently stained with a Goat anti-Mouse IgG (H+L) Highly Cross-Adsorbed Secondary Antibody and a Goat anti-Rabbit IgG (H+L) Highly Cross-Adsorbed Secondary Antibody for 2 h at room temperature. The cell nuclei were stained with DAPI. A fluorescence microscope was used for observation and for capturing images.

### 2.7. Western Blot Assay

After the model was established, the corneal tissues and HCECs of the four groups were lysed in a radioimmunoprecipitation assay lysis buffer containing 1% PMSF (Abmole, Houston, TX, USA) for protein extraction. Protein concentrations in the samples were detected by a BCA assay [24], and the detected proteins were diluted to the same concentration. Then, the protein samples were separated by sodium dodecyl sulfate polyacrylamide gel electrophoresis and transferred onto 0.2 mm polyvinyl difluoride membranes (Millipore, Schwalbach, Germany). The membranes were initially blocked with 5% BSA for 1 h at room temperature and then incubated with primary antibodies at 4 °C overnight. Subsequently, the membranes were treated with an enhanced chemiluminescence (ECL) reagent (Ecotop, Guangzhou, China), and then protein bands were detected by an ECL system. Quantification was performed using the ImageJ 1.8.0 software (NIH, Bethesda, MD, USA). Glyceraldehyde-3-phosphate dehydrogenase (GAPDH, Cell Signaling Technology, USA) was used as internal control. The primary antibodies used for the Western blot assay were Na^+^/K^+^-ATPase (Cell Signaling Technology, USA), E-cadherin (Abmart, Shanghai, China), α-SMA (Abcam, Waltham, MA, USA), vimentin (Santa Cruz, Dallas, TX, USA), Snail (Cell Signaling Technology, USA), TGF-β receptor 1 (TGF-βR1; Cell Signaling Technology, USA), Smad2/3 (Cell Signaling Technology, USA), and phosphorylated Smad2/3 (P-Smad2/3; Cell Signaling Technology, USA).

### 2.8. Statistical Analysis

The data presented in the figures are the results obtained in at least three replicate experiments and are presented as mean ± SD. The SPSS 22.0 software package (SPSS Inc., Chicago, IL, USA) was used for statistical analysis. Differences between groups were tested with a one-way analysis of variance (ANOVA). A *p*-value of <0.05 indicated statistical significance.

## 3. Results

### 3.1. SIRT1 Activation Ameliorated the Injury-Induced Corneal Endothelial Dysfunction in Rats

After mechanical injury of the corneal endothelium, corneal transparency and edema degree were evaluated using a slit lamp microscope. Corneal edema and opacity were most prominent 1 day after injury and then decreased gradually. Recovery was faster in the Test + RSV group than in the Test and Test + RSV + EX527 groups with the cornea becoming more transparent on days 3, 5, and 7 (Figure 1B).

The hematoxylin and eosin (H&E) staining results indicate a disorganized Descemet’s membrane and an increased thickness of the central cornea in the Test and Test + RSV + EX527 groups, whereas the Test + RSV group did not show such a significant thickening compared with the control group (Figure 1C,D). Overall, these results indicate that SIRT1 activation ameliorated the corneal endothelial dysfunction in the injury-induced corneal EnMT rat model.

### 3.2. SIRT1 Expression Was Downregulated in the Test Group of Rats

**Figure 2:**
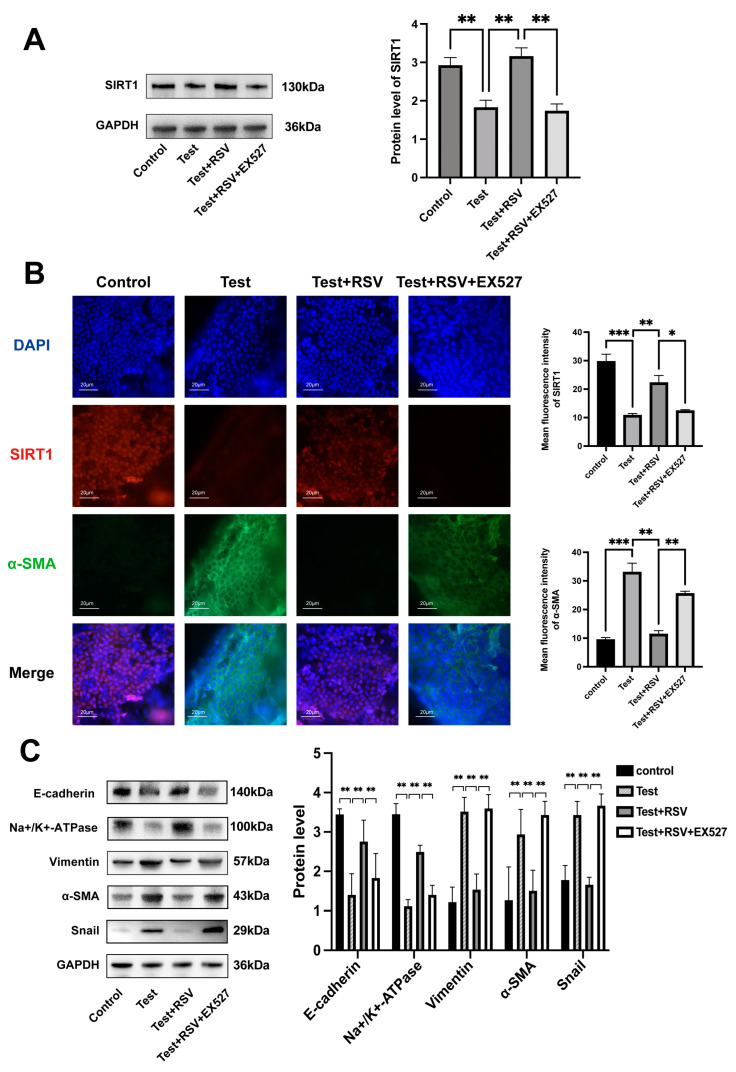
SIRT1 activation inhibited corneal EnMT in rats. (**A**) Protein level of SIRT1 in cornea. (**B**) Double immunofluorescence staining results for the expression levels of α-SMA (green) and SIRT1 (red) in corneal endothelium. Scale bar = 20 μm. (**C**) Western blot assay was performed to detect the expression levels of Na^+^/K^+^-ATPase, Snail, and E-cadherin and of the mesenchymal markers α-SMA and vimentin. GAPDH was used as internal control. * *p* < 0.05, ** *p* < 0.01, *** *p* < 0.001 *n* = 3.

Western blot assay and immunofluorescence staining were performed to detect the protein level of SIRT1 in rats after 7 days of treatment. In the Test group, the corneal endothelium of rats was mechanical injured. The results showed that SIRT1 was significantly downregulated in the Test group. By contrast, SIRT1 expression was upregulated by RSV, but this effect was counteracted by EX527 (Figure 2A,B).

### 3.3. RSV-Activated SIRT1 Inhibited Injury-Induced Corneal EnMT In Vivo

As mentioned before, EnMT has been found to occur in damaged corneal endothelium [2]. Thus, a Western blot assay was performed to evaluate the EnMT markers and endothelial function markers in vivo in the injury-induced corneal EnMT rat model (Figure 2C). In the Test group, the protein levels of α-SMA and vimentin (which are EnMT markers) and Snail (a transcriptional factor) were significantly increased, whereas the protein levels of the adhesion marker E-cadherin and the function marker Na^+^/K^+^-ATPase were downregulated. These changes were reversed by the treatment with RSV, which is an SIRT1 activator. However, the effect of RSV on EnMT was eliminated by the treatment with EX527, which is an SIRT1 inhibitor.

The immunofluorescence staining results also showed an increased protein expression of the mesenchymal marker α-SMA and a decreased expression of SIRT1 in both the Test and Test + RSV + EX527 groups, but these changes were reversed by RSV-activated SIRT1 (Figure 2B).

These results collectively proved that SIRT1 activation attenuated the injury-induced corneal EnMT in vivo.

### 3.4. SIRT1 Activation Inhibited TGF-β1-Induced HCEC Morphological Change and Migration

**Figure 3:**
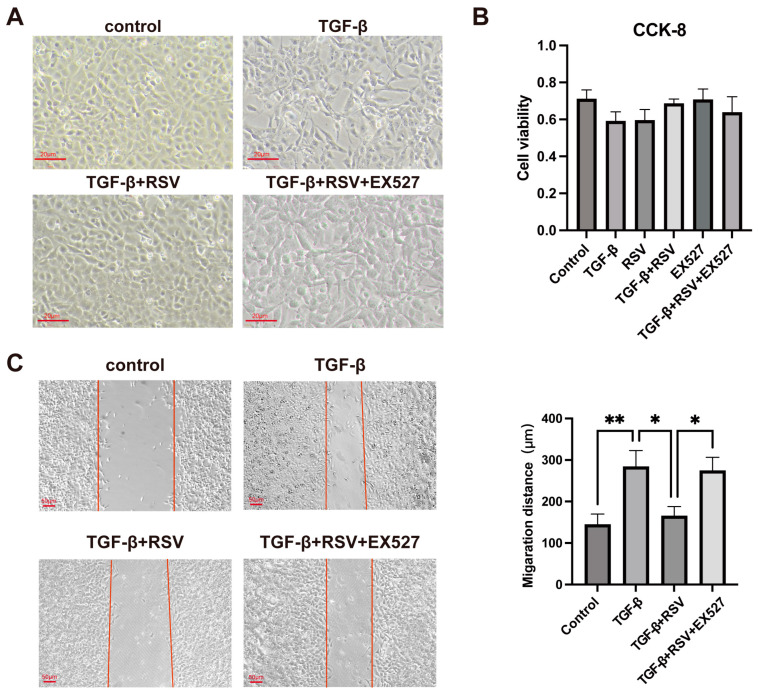
SIRT1 activation inhibited the migration of and the occurrence of morphological changes in TGF-β1-treated HCECs in vitro. (**A**) Morphological changes in HCECs. Scale bar = 100 μm. (**B**) Cell counting kit-8 assay was used to assess the cytotoxicity of different treatments toward HCECs. (**C**) Micrographs and statistical graph of HCEC migration in the scratch assay. Red lines indicate the migration edges. * *p* < 0.05, ** *p* < 0.01, *n* = 3. Scale bar = 100 μm. HCEC: Human Corneal Endothelial Cell.

TGF-β1 was used to induce EnMT in HCECs to evaluate the effect of SIRT1 on EnMT in vitro. The occurrence of EnMT following corneal endothelial injury induces fibrotic transformation, leading to morphological and functional changes in HCECs. In this study, TGF-β1 treatment of HCECs resulted in a change from having an oval cell morphology to having a spindle cell morphology. With the activation of SIRT1, such an effect was reversed in the TGF-β1 + RSV group wherein the HCECs showed a normal morphology. In the TGF-β1 + RSV + EX527 group, the HCECs also showed a spindle cell morphology because SIRT1 was inhibited by EX527 (Figure 3A).

EnMT enhances cell migration because of the degradation of the underlying basement membrane. The wound scratch assay results demonstrated enhanced TGF-β1-induced HCEC migration, but this effect was eliminated by RSV-activated SIRT1 (Figure 3C). In the TGF-β1 + RSV + EX527 group, the agonism of SIRT1 was inhibited by EX527 treatment, resulting in increased HCEC migration. Moreover, the CCK-8 test results showed no obvious difference in cell viability between each group, suggesting the absence of significant cellular cytotoxicity (Figure 3B). Our results showed that TGF-β1 promoted the migration ability of HCECs, but this effect was inhibited by the activation of SIRT1.

### 3.5. SIRT1 Expression Was Downregulated in the TGF-β1 Treated HCECs

**Figure 4:**
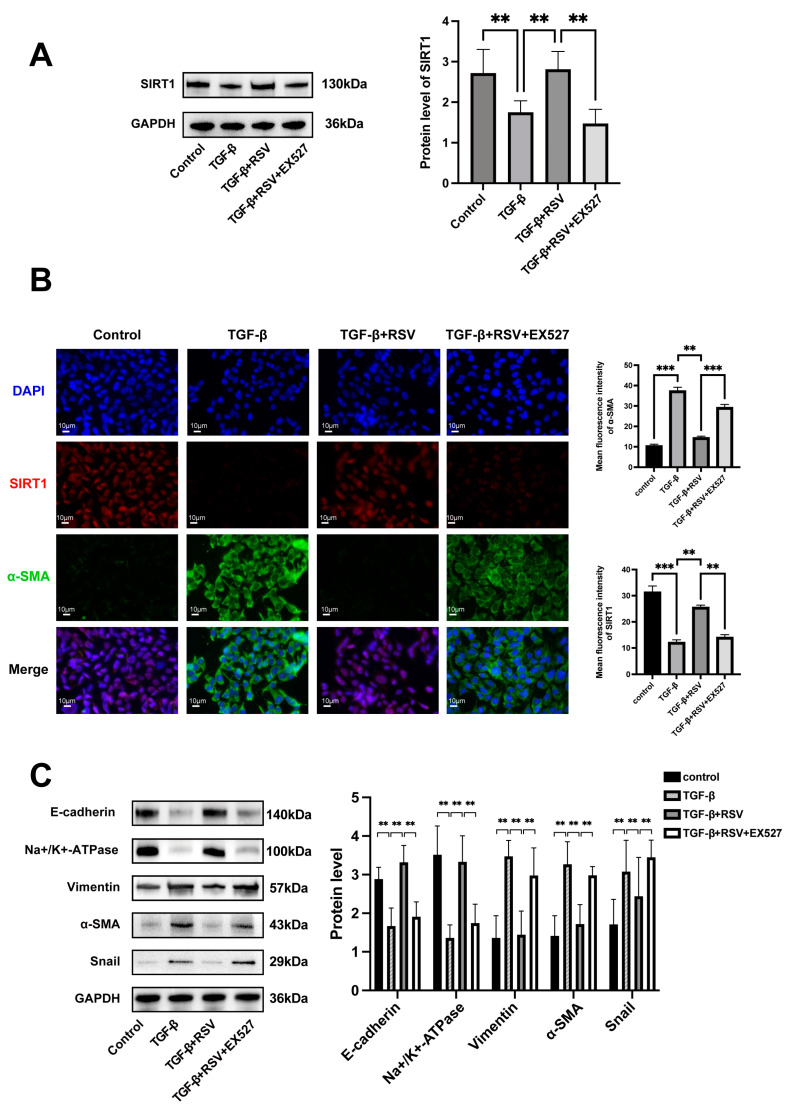
SIRT1 activation inhibited TGF-β1-induced EnMT in HCECs. (**A**) Protein level of SIRT1 in HCECs. (**B**) Double immunofluorescence staining results demonstrating the expression of α-SMA (green) and SIRT1 (red) in HCECs. Scale bar = 10 μm. (**C**) The protein levels of Na^+^/K^+^-ATPase, Snail, and VE-cadherin and the mesenchymal markers α-SMA and vimentin were evaluated by Western blot analysis. GAPDH was used as internal control. ** *p* < 0.01, *** *p* < 0.001, *n* = 3. HCEC: Human Corneal Endothelial Cell.

Western blot assay and immunofluorescence staining were performed to detect the protein level of SIRT1. SIRT1 was significantly downregulated in the TGF-β1 group. On the contrary, SIRT1 expression was upregulated by RSV, but this effect was counteracted by EX527 (Figure 4A,B). The results were consistent with the results of in vivo experiments.

### 3.6. SIRT1 Activation Alleviated the TGF-β1-Induced EnMT in HCECs

Western blot assay and double immunostaining were performed to detect the modulating effect of SIRT1 on corneal EnMT. Double immunofluorescence staining further verified the changes in SIRT1 and α-SMA levels (Figure 4B). The Western blot results showed that the protein expression levels of α-SMA, vimentin, and Snail were noticeably higher in the TGF-β1 and TGF-β1 + RSV + EX527 groups than in the control group (Figure 4C). In addition, the protein expression levels of E-cadherin and Na^+^/K^+^-ATPase significantly decreased. The TGF-β1-induced changes were inhibited by RSV-activated SIRT1 (Figure 4C). These results demonstrate that the activation of SIRT1 alleviated the TGF-β1-induced EnMT in HCECs.

### 3.7. SIRT1 Regulated the TGF-β1-Induced EnMT in HCECs via the TGF-β/Smad2/3 Pathway

**Figure 5:**
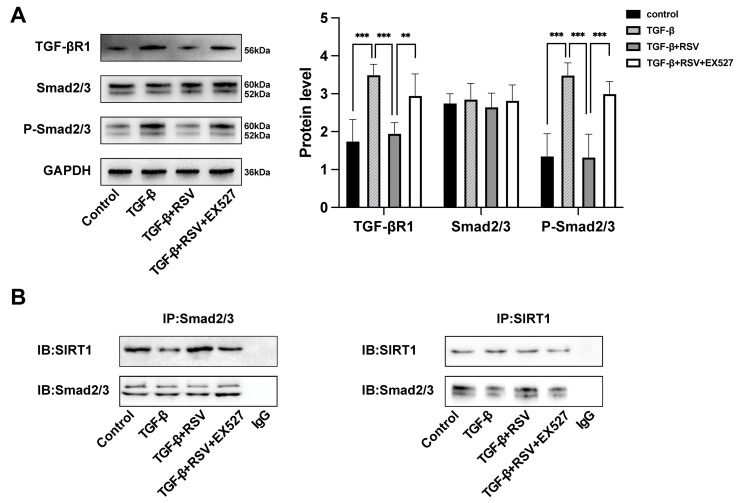
SIRT1 inhibited the expression of TGF-βR1 and P-Smad2/3 and interacted with Smad2/3 in TGF-β1-treated HCECs. (**A**) Western blot analysis of the TGF-β/Smad2/3 pathways. (**B**) Co-immunoprecipitation (CO-IP) results showing the interaction between SIRT1 and Smad2/3. ** *p* < 0.01, *** *p* < 0.001, *n* = 3.

As mentioned above, the TGF-β/Smad2/3 pathway is a target of SIRT1 [16]. To further investigate whether the TGF-β/Smad2/3 pathway plays a role in this regulatory process, we performed Western blot assays to analyze the protein levels of TGF-βR1, Smad2/3, and P-Smad2/3 in the TGF-β1-induced EnMT in HCECs. The Western blot data showed high protein levels of TGF-βR1 and P-Smad2/3 in the TGF-β1 and TGF-β1 + RSV + EX527 groups in vitro, but these results were reversed by RSV-activated SIRT1 in the TGF-β1 + RSV group (Figure 5A). Therefore, it can be inferred that the TGF-β/Smad2/3 pathway is involved in the regulation of corneal EnMT by SIRT1 in HCECs in vitro.

To further explore the specific mechanisms in this regulation process, we conducted a co-immunoprecipitation experiment in vitro involving HCECs, and the results confirmed the interaction between SIRT1 and Smad2/3 (Figure 5B). Overall, the results demonstrated that SIRT1 regulated the TGF-β1-induced EnMT via the TGF-β/Smad2/3 pathway.

## 4. Discussion

A normal number and quality of CECs maintain corneal transparency through a pumping mechanism, keeping the stroma in a slightly dehydrated state [9,25]. Slight corneal endothelial damage can be repaired by the migration of nearby normal cells; however, CECs usually undergo EnMT after being stimulated by factors such as surgical injury and persistent inflammation of the corneal stroma and anterior chamber, leading to abnormalities in both cell morphology and function [25,26]. When HCECs undergo EnMT in vivo, they secrete ECM that in turn form a retrocorneal membrane, resulting in decreased vision and even blindness [27,28,29]. Thus, EnMT is one of the reasons behind the structural and functional changes in CECs and the decrease in corneal transparency.

When cultured in vitro, HCECs are also prone to undergo EnMT, resulting in loss of normal function [30,31]. This phenomenon severely limits the possibility of clonal expansion of HCECs in vitro, making EnMT an obstacle in the development of tissue engineering applications involving HCECs for therapeutic purposes [32]. Therefore, our study aimed to elucidate the mechanism of EnMT in HCECs and identify ways to effectively reverse EnMT and preserve the normal phenotype and function of CECs, which may be an effective therapy for corneal diseases. SIRT1 is a histone deacetylase found in nuclei and cytoplasm; it is widely distributed in different organs of the human body and plays multiple physiological functions [33]. SIRT1 plays important roles in cell survival, apoptosis, tumor occurrence and development, anti-aging, and metabolic diseases [34,35]. Studies have shown that SIRT1 exerts a protective effect in cardiac and renal fibrosis [18]. In this experiment, we used RSV as an SIRT1 agonist given that it is the most potent activating compound for SIRT1 [36]. The results showed that RSV-activated SIRT1 reduced corneal edema and opacity in the rat corneal endothelial injury model. These effects were eliminated by treatment with EX527, which is an SIRT1 inhibitor.

EnMT is a complex biological process, the mechanism of which is still being investigated and involves the interaction of multiple molecules and pathways [37]. Most of the current research focuses on the role of key molecules and pathways in the TGF-β family and the application of their corresponding inhibitors [38,39,40]. TGF-β signaling involves a large family of multifunctional cytokines that are involved in regulation processes, such as cell proliferation, adhesion, and apoptosis [41,42,43]. TGF-β is an important cytokine for the repair of damages, and it exerts a chemotactic effect on the migration of myofibroblasts [44]. Studies have revealed that TGF-β is a critical factor in EnMT induction [45]. Another study has shown that when TGF-β was added to the culture medium of corneal stromal cells, overproduction of ECM by the cornea and cellular fibrosis could occur [46]. Similar results were obtained in our study. When TGF-β1 was added into the culture medium, the HCECs transformed into spindle-shaped cells, but this morphological transformation was inhibited by the activation of SIRT1.

Moreover, in the TGF-β1 group, the protein levels of the EnMT-associated molecules—namely, α-SMA, vimentin, and Snail—were upregulated, whereas the endothelial cell marker E-cadherin and the function marker Na^+^/K^+^-ATPase were downregulated. However, with RSV treatment, SIRT1 was activated, and the TGF-β1-induced effect was blocked, as demonstrated by the Western blot results for the TGF-β1 + RSV group. Similar western blot results were obtained in the injury-induced corneal EnMT model in rats. Together, these results suggest that the activation of SIRT1 attenuated the corneal EnMT in vivo and in vitro.

The process of EnMT in many organs involves multiple pathways, and the most well-characterized pathway is the TGF-β/Samd2/3 pathway [18,47,48]. Downstream transcription factors of TGF-β, Smad2, and Smad3 play a key role in TGF-β-induced tissue fibrosis and ECM production and in maintaining the activity of activated fibroblasts [16,49]. In the classical Smad pathway, TGF-β binds to and activates membrane receptors, leading to the phosphorylation of receptor-regulated Smad2 and Smad3, which in turn bind to Smad4, enter the nucleus, and then regulate the expression of EnMT-related genes [4,50,51]. Research has also shown that the TGF-β/Smad2/3pathway may be involved in the mechanism of corneal stromal fibrosis after Descemet stripping endothelial keratoplasty (DSEK) [52]. Our results show that the expression levels of TGF-βR1 and P-Smad2/3 were upregulated in injury-induced corneal EnMT, but this trend was reversed by the activation of SIRT1 by RSV. Similar results were obtained in HCECs in vitro. Moreover, the results of the co-immunoprecipitation experiment in vitro further demonstrated the interaction between SIRT1 and Smad2/3 in the EnMT regulation process. On the basis of these results, we speculate that SIRT1 inhibits EnMT progression through the TGF-β/Smad2/3 pathway, although the underlying mechanism needs further investigation.

Although TGF-β is recognized as the most important EnMT-inducing factor, many other pathways affect EnMT. For example, the Rho/ROCK signaling pathway is involved in human corneal EnMT [53], and Notch1 signaling exerts a protective effect toward adult coronary artery endothelial cells [54,55]. Moreover, some cytokines, such as interleukin-1β and tumor necrosis factor α, can cause cellular fibrosis during inflammation through EnMT [56,57,58]. Therefore, whether TGF-β is involved in SIRT1 regulation during EnMT through other pathways and whether other cytokines are involved in this process needs to be explored. The precise mechanism of SIRT1 in corneal EnMT also needs further investigation.

In summary, this research demonstrated that the activation of SIRT1 could attenuate injury-induced corneal EnMT in rats and TGF-β1-induced EnMT in HCECs. This study is the first to investigate the regulatory mechanism of SIRT1 in the EnMT of CECs both in vivo and in vitro. Moreover, our experimental results showed that RSV-activated SIRT1 could downregulate the expression levels of TGF-βR1 and P-Smad2/3 both in vivo and in vitro. Additionally, the interaction between SIRT1 and Smad2/3 was confirmed by co-immunoprecipitation in vitro. These results suggest that the TGF-β/Smad2/3 pathway is involved in this regulation process.

## Data Availability

Data are contained within the article.

## References

[1] He W., Zhang J., Gan T.Y., Xu G.J., Tang B.P. (2015). Advanced glycation end products induce endothelial-to-mesenchymal transition via downregulating Sirt 1 and upregulating TGF-beta in human endothelial cells. BioMed Res. Int..

[2] Li Z., Duan H., Li W., Jia Y., Zhang S., Zhao C., Zhou Q., Shi W. (2019). Nicotinamide inhibits corneal endothelial mesenchymal transition and accelerates wound healing. Exp. Eye Res..

[3] Xiang Y., Zhang Y., Tang Y., Li Q. (2017). MALAT1 Modulates TGF-β1-Induced Endothelial-to-Mesenchymal Transition through Downregulation of miR-145. Cell Physiol. Biochem.

[4] Li Z., Wang F., Zha S., Cao Q., Sheng J., Chen S. (2018). SIRT1 inhibits TGF-β-induced endothelial-mesenchymal transition in human endothelial cells with Smad4 deacetylation. J. Cell Physiol..

[5] Ubil E., Duan J., Pillai I.C., Rosa-Garrido M., Wu Y., Bargiacchi F., Lu Y., Stanbouly S., Huang J., Rojas M. (2014). Mesenchymal-endothelial transition contributes to cardiac neovascularization. Nature.

[6] Ribera J., Pauta M., Melgar-Lesmes P., Cordoba B., Bosch A., Calvo M., Rodrigo-Torres D., Sancho-Bru P., Mira A., Jimenez W. (2017). A small population of liver endothelial cells undergoes endothelial-to-mesenchymal transition in response to chronic liver injury. Am. J. Physiol. Gastrointest. Liver Physiol..

[7] Lee J.G., Kay E.P. (2012). NF-κB is the transcription factor for FGF-2 that causes endothelial mesenchymal transformation in cornea. Investig. Ophthalmol. Vis. Sci..

[8] Ho W.T., Chang J.S., Su C.C., Chang S.W., Hu F.R., Jou T.S., Wang I.J. (2015). Inhibition of matrix metalloproteinase activity reverses corneal endothelial-mesenchymal transition. Am. J. Pathol..

[9] Srinivas S.P. (2010). Dynamic regulation of barrier integrity of the corneal endothelium. Optom. Vis. Sci..

[10] Roy O., Leclerc V.B., Bourget J.M., Theriault M., Proulx S. (2015). Understanding the process of corneal endothelial morphological change in vitro. Investig. Ophthalmol. Vis. Sci..

[11] Gonzalez D.M., Medici D. (2014). Signaling mechanisms of the epithelial-mesenchymal transition. Sci. Signal..

[12] Sha L., Dong L., Lv L., Bai L., Ji X. (2015). HOXB9 promotes epithelial-to-mesenchymal transition via transforming growth factor-β1 pathway in hepatocellular carcinoma cells. Clin. Exp. Med..

[13] Pardali E., Sanchez-Duffhues G., Gomez-Puerto M.C., Ten Dijke P. (2017). TGF-beta-Induced Endothelial-Mesenchymal Transition in Fibrotic Diseases. Int. J. Mol. Sci..

[14] Tzavlaki K., Moustakas A. (2020). TGF-β Signaling. Biomolecules.

[15] Michan S., Sinclair D. (2007). Sirtuins in mammals: Insights into their biological function. Biochem. J..

[16] Liu Z.H., Zhang Y., Wang X., Fan X.F., Zhang Y., Li X., Gong Y.S., Han L.P. (2019). SIRT1 activation attenuates cardiac fibrosis by endothelial-to-mesenchymal transition. Biomed. Pharmacother..

[17] Cui Z., Zhao X., Amevor F.K., Du X., Wang Y., Li D., Shu G., Tian Y., Zhao X. (2022). Therapeutic application of quercetin in aging-related diseases: SIRT1 as a potential mechanism. Front. Immunol..

[18] Bugyei-Twum A., Ford C., Civitarese R., Seegobin J., Advani S.L., Desjardins J.F., Kabir G., Zhang Y., Mitchell M., Switzer J. (2018). Sirtuin 1 activation attenuates cardiac fibrosis in a rodent pressure overload model by modifying Smad2/3 transactivation. Cardiovasc. Res..

[19] Guarente L., Franklin H. (2011). Epstein Lecture: Sirtuins, aging, and medicine. N. Engl. J. Med..

[20] Jaliffa C., Ameqrane I., Dansault A., Leemput J., Vieira V., Lacassagne E., Provost A., Bigot K., Masson C., Menasche M. (2009). Sirt1 involvement in rd10 mouse retinal degeneration. Investig. Ophthalmol. Vis. Sci..

[21] Mimura T., Kaji Y., Noma H., Funatsu H., Okamoto S. (2013). The role of SIRT1 in ocular aging. Exp. Eye Res..

[22] Zheng T., Lu Y. (2011). Changes in SIRT1 expression and its downstream pathways in age-related cataract in humans. Curr. Eye Res..

[23] Karbasforooshan H., Karimi G. (2018). The role of SIRT1 in diabetic retinopathy. Biomed. Pharmacother..

[24] Olson B. (2016). Assays for Determination of Protein Concentration. Curr. Protoc. Pharmacol..

[25] Lee J., Jung E., Gestoso K., Heur M. (2020). ZEB1 Mediates Fibrosis in Corneal Endothelial Mesenchymal Transition Through SP1 and SP3. Investig. Ophthalmol. Vis. Sci..

[26] Lee J.G., Kay E.P. (2006). FGF-2-mediated signal transduction during endothelial mesenchymal transformation in corneal endothelial cells. Exp. Eye Res..

[27] Lee S.H., Kim K.W., Kim M.K., Chun Y.S., Kim J.C. (2014). Evaluation of stem cell components in retrocorneal membranes. J. Korean Med. Sci..

[28] Jakobiec F.A., Bhat P. (2010). Retrocorneal membranes: A comparative immunohistochemical analysis of keratocytic, endothelial, and epithelial origins. Am. J. Ophthalmol..

[29] Lee J., Jung E., Heur M. (2019). Injury induces endothelial to mesenchymal transition in the mouse corneal endothelium in vivo via FGF2. Mol. Vis..

[30] Hazra S., Sneha I.V., Chaurasia S., Ramachandran C. (2022). In Vitro Expansion of Corneal Endothelial Cells for Clinical Application: Current Update. Cornea.

[31] Senoo T., Joyce N.C. (2000). Cell cycle kinetics in corneal endothelium from old and young donors. Investig. Ophthalmol. Vis. Sci..

[32] Wang Y., Jin C., Tian H., Xu J., Chen J., Hu S., Li Q., Lu L., Ou Q., Xu G.T. (2022). CHIR99021 balance TGFbeta1 induced human corneal endothelial-to-mesenchymal transition to favor corneal endothelial cell proliferation. Exp. Eye Res..

[33] Sauve A.A., Wolberger C., Schramm V.L., Boeke J.D. (2006). The biochemistry of sirtuins. Annu. Rev. Biochem..

[34] Longo V.D., Kennedy B.K. (2006). Sirtuins in aging and age-related disease. Cell.

[35] Denu J.M., Gottesfeld J.M. (2012). Minireview series on sirtuins: From biochemistry to health and disease. J. Biol. Chem..

[36] Alcaín F.J., Villalba J.M. (2009). Sirtuin activators. Expert Opin. Ther. Pat..

[37] Lin F., Wang N., Zhang T.C. (2012). The role of endothelial-mesenchymal transition in development and pathological process. IUBMB Life.

[38] Chen X.F., Zhang H.J., Wang H.B., Zhu J., Zhou W.Y., Zhang H., Zhao M.C., Su J.M., Gao W., Zhang L. (2012). Transforming growth factor-β1 induces epithelial-to-mesenchymal transition in human lung cancer cells via PI3K/Akt and MEK/Erk1/2 signaling pathways. Mol. Biol. Rep..

[39] Díez M., Musri M.M., Ferrer E., Barberà J.A., Peinado V.I. (2010). Endothelial progenitor cells undergo an endothelial-to-mesenchymal transition-like process mediated by TGFbetaRI. Cardiovasc. Res..

[40] Reneker L.W., Bloch A., Xie L., Overbeek P.A., Ash J.D. (2010). Induction of corneal myofibroblasts by lens-derived transforming growth factor beta1 (TGFbeta1): A transgenic mouse model. Brain Res. Bull..

[41] Lichtman M.K., Otero-Vinas M., Falanga V. (2016). Transforming growth factor beta (TGF-β) isoforms in wound healing and fibrosis. Wound Repair Regen..

[42] Hu H.H., Chen D.Q., Wang Y.N., Feng Y.L., Cao G., Vaziri N.D., Zhao Y.Y. (2018). New insights into TGF-β/Smad signaling in tissue fibrosis. Chem Biol Interact.

[43] Okumura N., Kay E.P., Nakahara M., Hamuro J., Kinoshita S., Koizumi N. (2013). Inhibition of TGF-β signaling enables human corneal endothelial cell expansion in vitro for use in regenerative medicine. PLoS ONE.

[44] Huang Y., Wang Y., Wang X., Lin L., Wang P., Sun J., Jiang L. (2020). The Effects of the Transforming Growth Factor-β1 (TGF-β1) Signaling Pathway on Cell Proliferation and Cell Migration are Mediated by Ubiquitin Specific Protease 4 (USP4) in Hypertrophic Scar Tissue and Primary Fibroblast Cultures. Med. Sci. Monit..

[45] van Meeteren L.A., ten Dijke P. (2012). Regulation of endothelial cell plasticity by TGF-β. Cell Tissue Res..

[46] Toda M., Ueno M., Hiraga A., Asada K., Montoya M., Sotozono C., Kinoshita S., Hamuro J. (2017). Production of Homogeneous Cultured Human Corneal Endothelial Cells Indispensable for Innovative Cell Therapy. Investig. Ophthalmol. Vis. Sci..

[47] Ji H., Liu G., Han J., Zhu F., Dong X., Li B. (2020). C-phycocyanin inhibits epithelial-to-mesenchymal transition in Caski cells. Cancer Cell Int..

[48] Ma T.T., Meng X.M. (2019). TGF-β/Smad and Renal Fibrosis. Adv. Exp. Med. Biol..

[49] Dobaczewski M., Bujak M., Li N., Gonzalez-Quesada C., Mendoza L.H., Wang X.F., Frangogiannis N.G. (2010). Smad3 signaling critically regulates fibroblast phenotype and function in healing myocardial infarction. Circ. Res..

[50] Engel M.E., McDonnell M.A., Law B.K., Moses H.L. (1999). Interdependent SMAD and JNK signaling in transforming growth factor-beta-mediated transcription. J. Biol. Chem..

[51] Derynck R., Zhang Y.E. (2003). Smad-dependent and Smad-independent pathways in TGF-beta family signalling. Nature.

[52] Liu R., Li J., Guo Z., Chu D., Li C., Shi L., Zhang J., Zhu L., Li Z. (2023). Celastrol Alleviates Corneal Stromal Fibrosis by Inhibiting TGF-β1/Smad2/3-YAP/TAZ Signaling After Descemet Stripping Endothelial Keratoplasty. Investig. Ophthalmol. Vis. Sci..

[53] Wu Q., Ouyang C., Xie L., Ling Y., Huang T. (2017). The ROCK inhibitor, thiazovivin, inhibits human corneal endothelial-to-mesenchymal transition/epithelial-to-mesenchymal transition and increases ionic transporter expression. Int. J. Mol. Med..

[54] Li C., Dong F., Jia Y., Du H., Dong N., Xu Y., Wang S., Wu H., Liu Z., Li W. (2013). Notch signal regulates corneal endothelial-to-mesenchymal transition. Am. J. Pathol..

[55] Xie M., Zhang L., He C.S., Xu F., Liu J.L., Hu Z.H., Zhao L.P., Tian Y. (2012). Activation of Notch-1 enhances epithelial-mesenchymal transition in gefitinib-acquired resistant lung cancer cells. J. Cell. Biochem..

[56] Lee H.T., Lee J.G., Na M., Kay E.P. (2004). FGF-2 induced by interleukin-1 beta through the action of phosphatidylinositol 3-kinase mediates endothelial mesenchymal transformation in corneal endothelial cells. J. Biol. Chem..

[57] Adjuto-Saccone M., Soubeyran P., Garcia J., Audebert S., Camoin L., Rubis M., Roques J., Binétruy B., Iovanna J.L., Tournaire R. (2021). TNF-α induces endothelial-mesenchymal transition promoting stromal development of pancreatic adenocarcinoma. Cell Death Dis..

[58] Lee J.G., Ko M.K., Kay E.P. (2012). Endothelial mesenchymal transformation mediated by IL-1β-induced FGF-2 in corneal endothelial cells. Exp. Eye Res..

